# The Influence of Styrene Content in Solution Styrene Butadiene Rubber on Silica-Filled Tire Tread Compounds

**DOI:** 10.3390/polym15214288

**Published:** 2023-10-31

**Authors:** Gi-Yong Um, Taehoon Kwon, Seong Hwan Lee, Woong Kim, Jungsoo Kim, Hee Joong Kim, Jin Hong Lee

**Affiliations:** 1 Industrial Material Research Division, Korea Institute of Footwear and Leather Technology (KIFLT), Busan 47154, Republic of Korea; gyum@kiflt.re.kr (G.-Y.U.);; 2 School of Chemical Engineering, Pusan National University, Busan 46241, Republic of Korea; 3 Insulation Materials Research Center, Korea Electrotechnology Research Institute, Changwon 51543, Republic of Korea; 4 Department of Polymer Science and Engineering & Program in Environmental and Polymer Engineering, Inha University, Incheon 22212, Republic of Korea

**Keywords:** tire tread compound, solution styrene butadiene rubber, styrene, silica dispersion, filler–rubber interaction

## Abstract

In tire tread applications, achieving enhanced abrasion resistance, wet grip, and rolling resistance is crucial for optimizing overall performance. To realize improvements in these attributes for silica-filled tire tread compounds, it becomes imperative to improve the dispersity of silica filler by investigating the effect of each component in the tire tread compound. In this work, we study the effect of styrene content within solution styrene butadiene rubber (SSBR) on the properties of tire tread compounds. A higher styrene segment within SSBR contributes to increased silica dispersion and crosslink density. Thus, tire tread compounds featuring SSBR with increased styrene content not only improve physical and mechanical properties, but also enhance major characteristics tailored for tire tread applications. These findings provide valuable insights into advancing the reinforced performance of tire tread compounds through the strategic utilization of SSBR enriched in styrene content.

## 1. Introduction

The tire tread, which is in direct contact with the road surface, plays a central role within a tire due to its significant impact on crucial attributes. These include abrasion resistance (which affects tire durability), wet traction (which governs stability on wet surfaces), and rolling resistance (which has a substantial impact on fuel efficiency). These attributes collectively constitute the cornerstone of the “magic triangle” of tire performance [1,2,3]. Enhancing fuel efficiency in tires presents a formidable technical challenge, particularly in reducing rolling resistance during operation. The performance of rolling resistance is intricately linked to the energy dissipation characteristics, material volume, and distortion within the tire. The primary material within the tire, the filled-rubber compound, exhibits viscoelastic properties and contributes to energy losses due to cyclic deformations during driving. Among the tire’s components, the tread compound holds the largest volume fraction and plays a pivotal role in determining tire performance. Consequently, the primary focus of research by tire manufacturers striving to enhance rolling resistance performance has been on minimizing energy losses within the tread compound.

To overcome these challenges, researchers have been exploring methods to reduce rolling resistance by reducing tire weight. In essence, tire weight reduction is achieved by decreasing tread depth. A fundamental approach to achieving this reduction involves enhancing the tread compound’s abrasion resistance. Consequently, it becomes possible to simultaneously utilize both silica and carbon black to improve viscoelastic properties while achieving weight reduction through reduced tread depth. Additionally, research is ongoing regarding modifications to the curing conditions to enhance the rolling resistance performance of tires. These curing conditions significantly impact the physical properties of vulcanizates, and extensive investigations into the effects of temperature on the chemical structure of vulcanizates are being conducted. Generally, lowering the curing temperature leads to an increase in the number of poly-sulfide structures, consequently enhancing viscoelastic properties.

In 2012, Europe implemented a tire labeling system that enforces regulations governing rolling resistance, wet grip, and pass-by noise performance. In light of recent concerns regarding tire road wear particles, it is expected that additional regulations addressing wear performance will be introduced. When assessing the qualities of tread compounds, the rolling performance of a passenger car radial tire (PCR) benefits from reduced energy loss. Conversely, wet grip performance improves as energy loss increases. Consequently, most studies on PCR tire tread compounds aimed at enhancing rolling performance have focused on reducing energy loss while minimizing any potential degradation of wet grip performance. The introduction of the silica–silane system has notably enhanced the rolling resistance performance of tread compounds, which are traditionally reliant on carbon black as a filler, and has yielded significant research outcomes that effectively address the trade-off between energy loss reduction and wet grip performance. Furthermore, it is well documented that the formation of composites between inorganic materials such as silica and organic materials results in heightened toughness, rigidity, and thermal stability of these composites [4,5,6,7].

The increasing emphasis on environmental sustainability in contemporary times has significantly shaped the landscape of tire tread compounds. For example, in recent years, the EU has introduced stringent regulations aimed at curtailing greenhouse gas emissions, with a specific focus on carbon dioxide. Passenger cars are major contributors, accounting for approximately 13% of the total CO_2_ emissions within the EU. Given that the tread of a passenger car tire plays a substantial role in a vehicle’s fuel consumption due to its impact on rolling resistance, the development of novel tire tread formulations assumes paramount importance in the endeavor to reduce the carbon footprint. As of 2021, new passenger cars are required to achieve a 27% reduction in CO_2_ emissions compared to 2015 levels. Following the advent of the “Green tire,” precipitated silica has emerged as a compelling alternative to carbon black fillers for manufacturing high-performance automotive tires [8,9,10]. Utilizing silica as a reinforcement agent enhances the overall performance of tire treads, outperforming compounds containing carbon black fillers. Silica exhibits low hysteresis at tire rolling frequencies, typically in the range of 10–10^3^ Hz. Also, silica shows higher hysteresis at wet traction frequencies of 10^5^–10^6^ Hz in tire tread composites when compared to carbon black. Notably, this enhancement yields exceptional attributes, including reduced rolling resistance, minimized heat build-up, and enhanced wet grip characteristics [11,12,13].

Effective rubber reinforcement depends on achieving an optimal degree of filler dispersion. Furthermore, the interaction between the filler and the rubber plays a pivotal role in the desired reinforcement, which is driven by both chemical and physical mechanisms. As such, the inclusion of a compatibilizer, namely a silane coupling agent, becomes a fundamental component in the design of silica-filled rubber compounds. The silane coupling agent facilitates chemical interactions between silica and rubber during both the mixing and vulcanization processes. This interaction, often referred to as silanization, significantly reduces the polarity of silica, resulting in enhanced processability and improved filler dispersion. Post-silanization, a subsequent reaction occurs between the silane and the rubber polymer, known as filler-polymer coupling. This further reinforces the material, albeit with a potential drawback in terms of processability, depending on the chemical structure of the silanes and the occurrence of pre-scorch.

However, due to the presence of numerous hydrophilic polar silanol groups on its surface, silica exhibits poor compatibility with the hydrophobic non-polar rubber matrix [14]. Consequently, achieving effective dispersion becomes challenging, with a tendency for agglomeration due to strong filler–filler interactions [15,16]. Furthermore, the formation of strong hydrogen bonds with vulcanization accelerators results in their adsorption, leading to the formation of silica aggregates through the potent bonding forces between silica particles [17]. This adversely impacts silica dispersion and subsequently alters the mechanical properties. Consequently, the need to enhance silica dispersion as a reinforcing agent while optimizing filler–rubber interactions becomes imperative to enhance the magic triangle performance of tread compounds [18,19,20,21]. Nevertheless, there is a limited body of research focusing on the direct investigation of the individual component effects within tire tread compounds featuring silica dispersion.

This study aims to investigate the influence of styrene content within solution styrene butadiene rubber (SSBR) on both silica dispersion and tire tread performance. This investigation focuses on understanding how the variation in styrene content affects these factors. Indirect and direct methods were employed to assess the dispersity of silica particles: Payne effect analysis provided indirect confirmation, while the observation of compound morphology through the SEM offered direct insights. Concurrently, the processed compound underwent evaluation for vulcanization properties, crosslink density, mechanical attributes, abrasion resistance, and dynamic viscoelastic properties. The findings from this study are expected to provide valuable guidance for enhancing silica dispersion and thereby optimizing the resulting performance within tire tread compounds through targeted alterations of a single compound segment.

## 2. Materials and Methods

### 2.1. Materials

Three distinct SSBRs were employed, each with varying styrene content: 6270M, C 6450SL, and 6431H, all of which were provided by Kumho Petrochemical Co., Daejeon, Republic of Korea. Comprehensive information regarding the specific attributes of these SSBR variants is outlined in Table 1. The reinforcing filler for the tire tread compound consisted of precipitated silica (Z175MP, Solvay SA/NV, Brussels, Belgium) with a BET surface area of 175 m^2^ g^−1^. Bis-[(triethoxysilyl)propyl]tetrasulfide (TESPT; Si-69, Nanjing Aocheng Chemical Co., JiangSu, China) was employed as the silane coupling agent. Zinc oxide (ZnO), stearic acid (StA), and polypropylene glycol 4000 (PEG 4000) were used as vulcanization activators. Sulfur, *n*-cyclohexyl-2-benzothiazole sulfonamide (CBS), and 1,3-diphenyl-guadinine (DPG) were used as crosslinking agents and cure accelerators. The quantitative evaluation of crosslink density was facilitated using toluene (99.8%), acetone (99.7%), tetrahydrofuran (THF, 99.8%), and *n*-hexane (95%) obtained from Samchun Pure Chemical Co., Seoul, Republic of Korea.

### 2.2. Sample Preparation

The compound was prepared through a two-step process, namely the silica master batch (SMB) mixing and the final master batch (FMB) steps. In the initial SMB phase, three SSBR compounds were generated within a closed internal mixer (kneader, 300 cc, Moriyama, Japan). The process commenced with the introduction of 100 phr of SSBR into the mixer, which underwent mastication for 1 min at a rotor speed of 30–35 rpm while maintaining a mixing temperature of 80 °C. Then, 5 phr of ZnO, 1 phr of StA, and 3 phr of PEG 4000 were introduced into the SSBR, and mixed for another 1 min. Subsequently, 60 phr of silica and 6 phr of silane coupling agent were incorporated, and the mixture was further blended for 13 min. During the compounding procedure, silica and the silane coupling agent were added in four divided portions during this mixing procedure. The detailed compounding formulation and procedure are depicted in Table 2 and Table 3, respectively. The final dump temperature was set between 120 and125 °C. The resulting SMB compounds were then combined with sulfur and vulcanization accelerators to fabricate FMB compounds. The combination took place through an 8-inch two-roll mill for 5 min, followed by sheeting. After that, the FMB was vulcanized in a heating press at 160 °C using the optimum vulcanization time determined from the vulcanization characterization.

### 2.3. Characterization

To assess the curing characteristics, the sheeted FMBs underwent torque measurement at a temperature of 160 °C for a duration of 30 min. This evaluation was conducted using a rubber process analyzer (RPA elite, TA Instruments, New Castle, DE, USA) with an oscillating frequency of 1.667 Hz, in accordance with ASTM D 2084 standards. The cure curves yielded insights into key parameters, such as the minimum and maximum torque values, as well as the optimal curing time.

The dispersion of the silica filler within the SSBR compounds was ascertained through a combination of Payne effect analysis and the observation of morphology on the fractured surfaces using field-emission scanning electron microscopy (FE-SEM; S4800, Hitachi, Tokyo, Japan). Payne effect analysis was executed utilizing the rubber process analyzer with a strain range spanning from 0.1% to 100% and a frequency of 1.0 Hz at a temperature of 60 °C. The assessment involved calculating the difference in storage modulus between the initial and final points to quantify the Payne effect exhibited by the samples.

For the quantitative analysis of crosslink density, the vulcanized rubbers were cut into specimens with 10 mm × 10 mm × 2 mm and were initially weighed prior to the extraction process. To eliminate organic components, the samples underwent a 48 h immersion in THF followed by another 48 h immersion in *n*-hexane, both conducted at room temperature. Subsequently, the samples were air-dried for 48 h at room temperature and then re-weighed post-extraction. These dried specimens were further subjected to a 24 h swelling in toluene, allowing for subsequent measurement of the weight of the swollen specimens, thereby enabling the computation of the crosslinking density. Equation (1) was used to calculate the volume fraction value using the measured weight value.

(1) $$V_{r}=\frac{\frac{W_{d}-W_{r}}{\rho_{r}}}{\frac{W_{d}-W_{r}}{\rho_{r}}+\frac{W_{s}-W_{d}}{\rho_{s}}}$$

where *V*_r_ is the volume fraction of rubber in the swollen gel at equilibrium, *W*_d_ is the weight of the dried sample, *W*_f_ is the weight of the filler in the sample, *W*_s_ is the weight of the swollen sample, ρ_s_ is the density of solvent, and ρ_r_ is the density of rubber.

The acquired *V*_r_ value was used in Equation (2), the Flory–Rehner equation, to determine the quantitative crosslink density [22,23].

(2) $$\nu =\frac{1}{2M_{c}}=-\frac{ln(1-V_{r})+V_{r}+\chi {V_{r}}^{2}}{2\rho_{r}V_{s}({V_{r}}^{1/3}-\frac{V_{r}}{2})}$$

where *ν* (mol g^−1^) is the crosslink density, M_C_ (g mol^−1^) is the average molecular weight between crosslink points, *V*_r_ is the volume fraction of rubber in the swollen gel at equilibrium from Equation (1), *V*_s_ (cm^3^ mol^−1^) is the molar volume of solvent, ρ_r_ (g cm^−3^) is the density of the rubber sample, and χ is the polymer–solvent interaction parameter.

The mechanical properties of the vulcanized rubber compounds were evaluated through the creation of dumbbell-shaped specimens, adhering to the guidelines specified by ASTM D 412. The evaluation encompassed the determination of the modulus, tensile strength, and elongation at the point of fracture. Utilizing a universal testing machine (UTM; 3345, Instron, Norwood, MA, USA) with a 500 N load cell, mechanical tests were conducted at room temperature using a crosshead speed of 500 mm/min. The hardness of the vulcanizate was determined by employing a Shore A durometer (JIS K 6253, Asker, Kyoto, Japan) following ASTM D 2240 guidelines. The measurement involved pressing the specimen after overlapping it to a thickness of 6 mm.

The evaluation of abrasion resistance was conducted via the DIN abrasion test, in accordance with ASTM D 5963 standard. For this test, cylindrical specimens were prepared specifically for the DIN abrasion test, featuring a diameter of 16 mm and a thickness of 8 mm. The actual test was executed utilizing a DIN abrasion tester (Withlab, Gunpo-si, Gyeonggi-do, Republic of Korea), involving the application of an abrasive cloth mounted on the cylindrical apparatus. The specimen underwent abrasion for a distance of 40 m, subjected to a vertical load of 5 N and a drum rotation of 40 ± 1 rpm.

The dynamic viscoelastic characteristics of the compounds were investigated using a temperature sweep test conducted with a dynamic mechanical analyzer (DMA; ARES-G2, TA Instruments, New Castle, DE, USA). The glass transition temperature (T_g_), peak tan δ, and tan δ at 0 °C and 60 ℃ of the vulcanizates were ascertained by gradually increasing the temperature from −80 °C to 80 °C at a rate of 3 °C min^−1^ and a strain of 0.2%, all while maintaining temperature sweep conditions and employing a frequency of 10 Hz.

## 3. Results and Discussion

**Figure 1:**
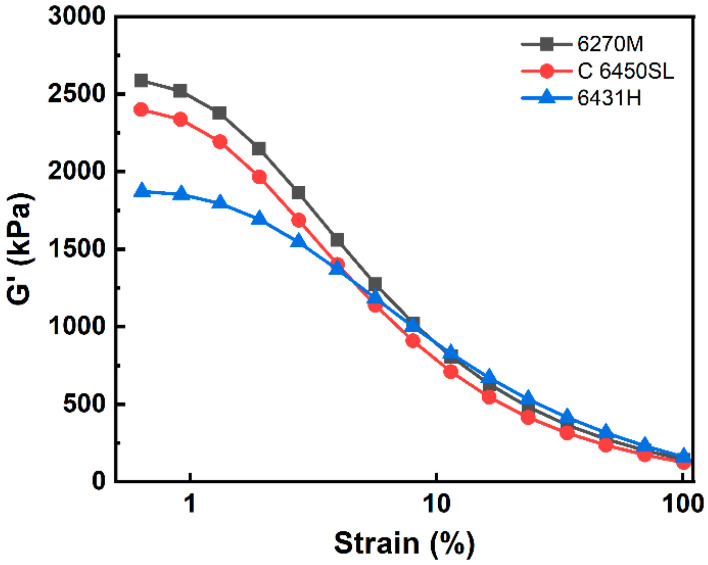
Payne effect in the tire tread compounds before vulcanization.

In the silica-filled tire tread compound, the dispersity of silica particles and the filler–rubber interaction are pivotal factors influencing both the physical/mechanical properties and the tire tread performance. These factors can be confirmed indirectly through the analysis of the Payne effect. Storage modulus (G′) vs. strain curves and precise values of three tire tread compounds are presented in Figure 1 and Table 4. The storage modulus at the initial point serves as an indicator of filler–filler interaction, while the difference in storage modulus (∆G′) between the starting and ending points provides insight into the filler–rubber interaction. A smaller ∆G′ value signifies a more favorable filler–rubber interaction [24,25,26]. The ∆G′ values of the tire tread compounds based on 6270M, C 6450SL, and 6431H are 2442, 2276 and 1712 kPa, respectively. The compound featuring SSBR 6270M exhibits increased filler–filler interaction, possibly due to the agglomeration of silica particles, along with a reduced filler–rubber interaction. In contrast, the SSBR 6431H-based compound displays reduced filler–filler interaction, attributed to lower agglomeration, coupled with increased filler–rubber interaction. This behavior may be attributed to the rigid and electron-rich aromatic styrene group present in SSBR, which hinders the agglomeration of silica particles. Therefore, it is plausible to conjecture that a higher styrene content within the SSBR contributes to improved dispersity of silica particles and consequent filler–rubber interaction within the tire tread compound.

**Figure 2:**
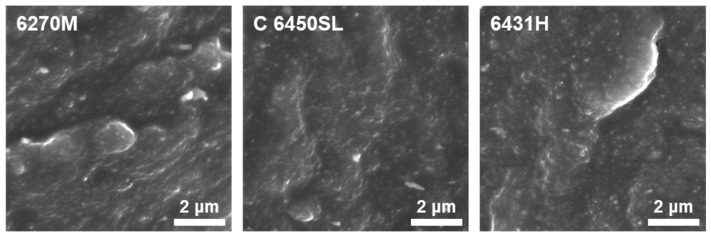
Cross-sectional SEM micrographs of the tire tread compounds.

Direct confirmation of silica filler dispersity was achieved by examining the cross-sectional morphology of the tire tread compound using the SEM. In Figure 2, the SEM images of the cross-sectional morphology of each compound are presented. The image of the 6270M-based compound reveals larger agglomerations within the rubber matrix, indicating poor silica particle dispersity. The micrograph of the C 6450SL-based compound shows relatively smaller agglomerations compared to the 6270M-based compound. In contrast, the 6431H-based compound exhibits significantly smaller agglomerations and well-dispersed silica particles within the compound. This observation aligns with the results of the Payne effect analysis and emphasizes that compounds with higher styrene content in SSBR exhibit improved silica particle dispersity.

**Figure 3:**
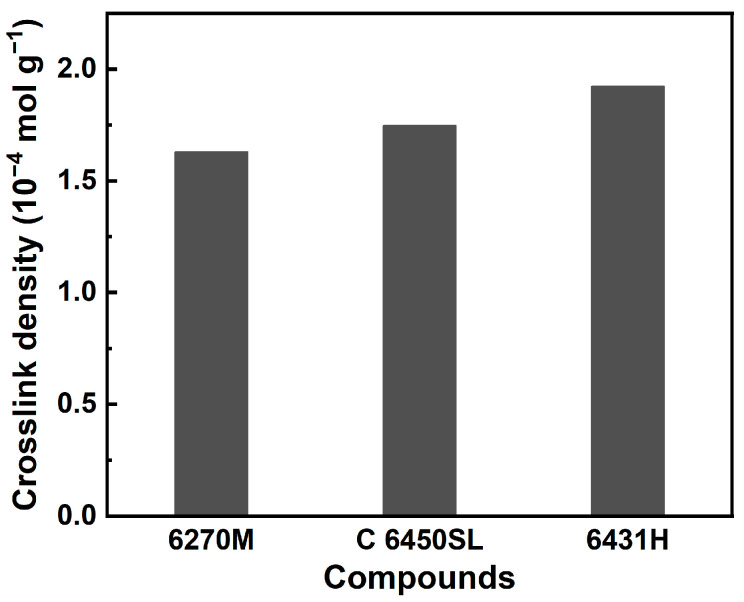
Crosslink density of the tire tread compounds.

The crosslink density of the three SSBR compounds is presented in Figure 3 and detailed in Table 5. The crosslink density values of 6270M-, C 6450SL-, and 6431H-based tire tread compounds are 1.629 × 10^−4^, 1.747 × 10^−4^, and 1.923 × 10^−4^ mol g^−1^, respectively. Notably, the crosslink density values of the three compounds were found to be similar. This observation suggests that crosslink density is influenced more by factors beyond the styrene content present in the SSBR. Nonetheless, specific differences in the values of crosslink density were observed across the compounds. Interestingly, the compound employing SSBR with a higher styrene content displayed an elevated crosslink density value. The observed increase in crosslink density can be attributed to the higher content of styrene groups featuring electron-rich aromatic rings. This outcome implies that a higher styrene content in the SSBR is conducive to enhanced mechanical properties and improved performance of tire tread compound.

**Figure 4:**
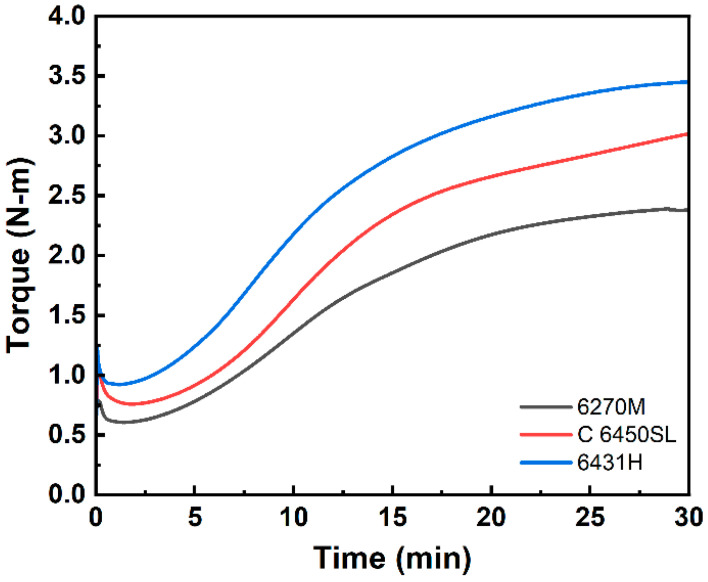
Cure curves of the tire tread compounds.

The cure characteristics of the three SSBR compounds during vulcanization are depicted in Figure 4 and detailed in Table 6. The differences in torque between the maximum (T_max_) and minimum (T_min_) values (∆T) in the compounds based on 6270M, C 6450SL, and 6431H SBR are 1.78, 2.26 and 2.53 N-m, respectively. ∆T is found to be lowest in the compound based on 6270M and highest in the compound based on 6431H. It is established that ∆T correlates with both crosslink density and the dispersion of silica [27,28,29,30]. Furthermore, the T_max_ and ∆T values exhibit a consistent pattern with the Mooney viscosity of the respective SSBR compounds, as outlined in Table 1. Consequently, the styrene content within the SSBR is evidently influential in enhancing crosslink density, thereby leading to improved mechanical properties.

**Figure 5:**
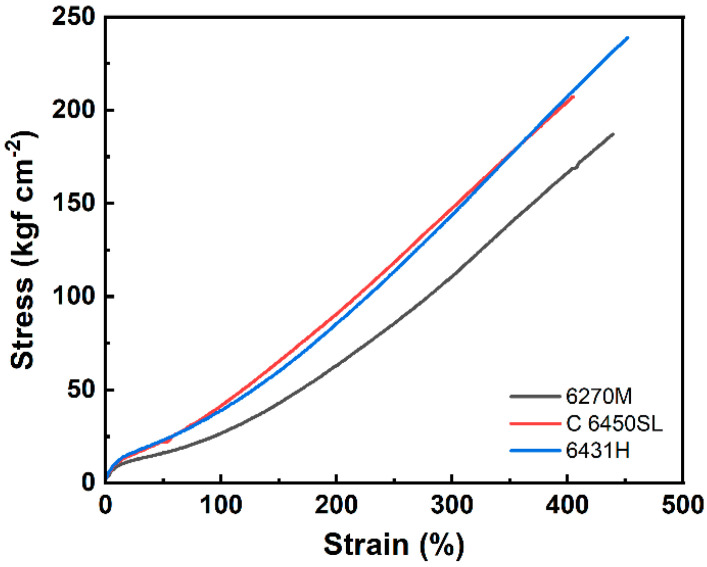
Stress–strain curves of the tire tread compounds.

The mechanical properties of the three tire tread compounds are illustrated in Figure 5 and detailed in Table 7. The Shore A hardness values of the 6270M-, C 6450SL-, and 6431H-based compounds are 74, 75, and 76, respectively. Hardness exhibited a slight increase in styrene content within the SSBR. This outcome can be attributed to the presence of the rigid styrene segment within the SSBR main chain [31]. Moreover, noteworthy enhancements were observed in the 300% modulus (111, 148, and 144 kgf cm^−2^, respectively), tensile strength (187, 208, and 239, respectively), and tear strength (62.0, 80.7, and 81.5 kgf cm^−1^, respectively) of the compounds. These improvements in mechanical properties can be attributed to the concurrent increase in the content of the rigid styrene and the elevation in crosslink density.

Table 8 presents the results of the DIN abrasion test measurements conducted on the three SSBR compounds. The volumetric DIN abrasion loss values of the tire compounds with 6270M, C 6450SL, and 6431H SBR show 168, 147, and 134 mm^3^, respectively. The DIN abrasion performance of tire treads is subject to the influence of numerous factors, including glass transition temperature (T_g_), crosslink density, the dispersion of silica particles, and the interaction between the filler and rubber components [32,33,34]. As illustrated in Table 8, an increase in the styrene content of the SSBR leads to elevated DIN abrasion resistance. As previously mentioned, the tire tread compound formulated with the SSBR possessing the highest styrene content (6431H) demonstrated notable inprovements in filler–rubber interaction, silica dispersion, and crosslink density. Consequently, the enhanced abrasion resistance observed in the 6431H-based compound stems from a combination of these enhanced factors attributed to the elevated styrene content.

**Figure 6:**
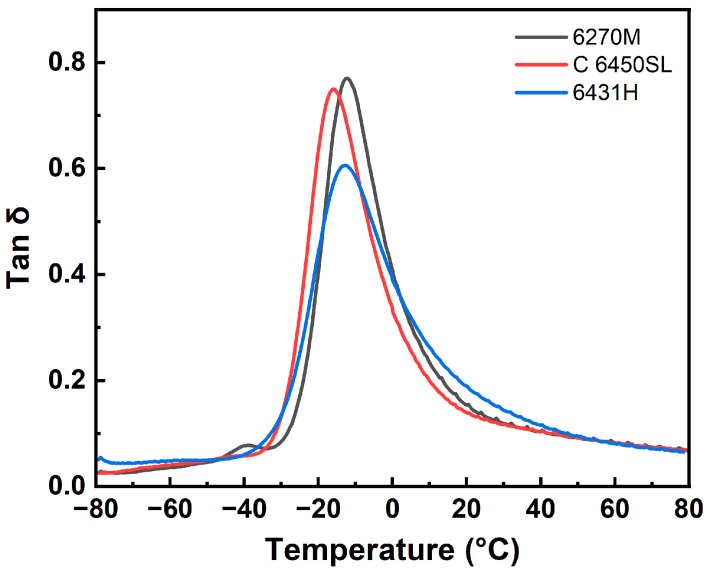
Tan δ curves of the tire tread compounds.

Figure 6 and Table 9 show the dynamic viscoelastic properties of the three SSBR compounds. The peak tan δ values at T_g_ of the three compounds (−12.08, −16.20, and −12.91 °C, respectively) exhibited a decline with the increase in styrene content within the SSBR. This trend can be ascribed to the heightened crosslink density stemming from the presence of the electron-rich styrene group [35]. The tan δ value at 0 °C is typically considered an indicator of wet grip performance within tire tread compounds [36,37]. The tan δ values at 0 °C of the three compounds are 0.4068, 0.3300, and 0.3883, respectively. The outcomes revealed that the tan δ values at 0 °C are more closely linked to the T_g_ of tire tread compounds rather than the styrene content within the SSBR. Consequently, wet grip performance is relatively similar between the 6270M-based and 6431H-based compounds, while distinctly lower values are observed in the compound utilizing C 6450SL SSBR. Conversely, a lower tan δ value at 60 °C is indicative of superior rolling resistance performance within tire tread compounds [38,39]. The tan δ values at 60 °C of the three compounds are 0.0823, 0.0818, and 0.0805, respectively. The tan δ values of the three compounds at 60 °C exhibit a decrease with the increase in styrene content. This outcome is attributed to the augmented crosslink density and improved silica dispersion within the compound featuring higher styrene content in the SSBR. As a result, the SSBR characterized by a higher styrene content emerges as a suitable candidate for tire tread applications, offering improved performance.

## 4. Conclusions

In summary, this study investigated the impact of styrene content within SSBR on silica-filled tire tread applications. The presence of higher styrene content within SSBR led to marked improvements in both silica dispersion and filler–rubber interaction. These enhancements were attributed to the rigid and electron-rich styrene segment within the SSBR main chain, which was validated through Payne effect analysis and SEM morphology observation. Furthermore, the electron-rich styrene segment contributed to an increase in the crosslink density of vulcanized rubber compounds. Consequently, the tire tread compound incorporating higher styrene content demonstrated enhanced physical and mechanical properties, along with improved performance attributes such as abrasion resistance and rolling resistance. These findings highlight the potential of SSBR with elevated styrene content as a compelling candidate for high-performance silica-filled tire tread applications.

## Figures and Tables

**Table 1:** Characteristics of SSBRs.

	Styrene Content (%)	Vinyl Content (%, in Butadiene)	Mooney Viscosity	T_g_ (°C)
6270M	25	63	51	−30
C 6450 SL	35	40	53	−36
6431H	40	35	62	−30

**Table 2:** Compounding formulation (unit: phr).

		6270M	C 6450SL	6431H
Stage 1 (SMB Mixing)	SSBR (6270M)	100	-	-
	SSBR (C 6450SL)	-	100	-
	SSBR (6431H)	-	-	100
	Silica	60		
	TESPT	6		
	ZnO	5		
	StA	1		
	PEG 4000	3		
Stage 2 (FMB Mixing)	Sulfur	0.5		
	CBS	1.5		
	DPG	1.5		

Notes: phr—parts per hundred rubber.

**Table 3:** Compounding procedure.

Step	Time (min)	Action
1st step (mixing in a kneader)	0:00	Add rubber (80 °C)
	1:00	Add ZnO + StA + PEG 4000
	2:00	Add silica + TESPT (1/4)
	5:00	Add silica + TESPT (2/4)
	8:00	Add silica + TESPT (3/4)
	11:00	Add silica + TESPT (4/4)
	15:00	Dump (120–125 °C)
2nd step (mixing in a two-roll mill)	0:00	Add SMB compound
	1:00	Add sulfur + CBS + DPG
	5:00	Dump

**Table 4:** Storage modulus (G′) values of the tire tread compounds.

	6270M	C 6450SL	6431H
G′ at initial point (kPa)	2585	2400	1871
G′ at final point (kPa)	143.0	123.7	159.3
∆G′ (kPa)	2442	2276	1712

**Table 5:** Crosslink density values of the tire tread compounds.

	6270M	C 6450SL	6431H
Crosslink density (10^−4^ mol g^−1^)	1.629	1.747	1.923

**Table 6:** Cure characteristics result of the tire tread compounds.

	6270M	C 6450SL	6431H
t_10_ (min)	5.03	5.72	4.52
t_90_ (min)	20.94	23.60	20.79
T_min_ (N-m)	0.61	0.76	0.92
T_max_ (N-m)	2.39	3.02	3.45
∆T (N-m)	1.78	2.26	2.53

**Table 7:** Mechanical properties of the tire tread compounds.

	6270M	C 6450SL	6431H
Hardness (Shore A)	74	75	76
300% modulus (kgf cm^−2^)	111	148	144
Elongation at the break (%)	440	410	450
Tensile strength (kgf cm^−2^)	187	208	239
Tear strength (kgf cm^−1^)	62.0	80.7	81.5

**Table 8:** DIN abrasion weight loss of the tire tread compounds.

	6270M	C 6460SL	6431H
DIN abrasion loss (mg)	203	178	164
DIN abrasion loss (mm^3^)	168	147	134

**Table 9:** Viscoelastic properties of the tire tread compounds.

	6270M	C 6450SL	6431H
T_g_ (°C)	−12.08	−16.20	−12.91
Peak tan δ	0.7704	0.7494	0.6057
Tan δ at 0 °C	0.4068	0.3300	0.3883
Tan δ at 60 °C	0.0823	0.0818	0.0805

## Data Availability

The data presented in this study can be obtained upon request from the corresponding author.
